# Odor alters color preference in a foraging jumping spider

**DOI:** 10.1093/beheco/ary068

**Published:** 2018-05-23

**Authors:** Michael E Vickers, Lisa A Taylor

**Affiliations:** 1 Entomology and Nematology Department, University of Florida, Gainesville, FL, USA; 2 Florida Museum of Natural History, University of Florida, Gainesville, FL, USA

**Keywords:** aposematism, color, Habronattus, multimodality

## Abstract

In many prey taxa with aposematic coloration, prey defenses also involve signals in other modalities (odors, sounds, etc.), yet the selective forces that have driven multimodality in warning displays are not well understood. One potential hypothesis that has recently received support in the avian literature (but has yet to be examined in invertebrates) is that different signal components may interact synergistically, such that one component of a signal (odor) may trigger a predator’s aversion to another component of a signal (color). Here, we gave jumping spiders (*Habronattus trimaculatus*) the choice between red or black prey (artificially colored termites) in either the presence or absence of odor from the chemically defended coreid bug (*Acanthocephala femorata*). When the odor was present, spiders were more likely to avoid the color red compared with when the odor was absent. Interestingly, this pattern only held up when the odor was novel; subsequent exposure to the odor had no effect on color preference. Moreover, this pattern only held for the color red (a color typically used as a warning color and often paired with odor). We replicated this experiment giving spiders the choice between green or black prey, and found that the presence of the odor had no effect on the spiders’ responses to the color green. We discuss these findings in the context of predator psychology and the evolution of prey coloration.

## Teaser

Many animals use both bright colors and noxious odors to keep predators away, yet it is not clear why such combinations of colors and odors are so effective. Here, we show that voracious jumping spiders are more likely to avoid prey of certain colors (specifically, red prey) when an aversive odor is present. This may help explain why certain colors and odors frequently evolve together.

## INTRODUCTION

Predator psychology has long been recognized as an important selective force driving the evolution of prey defenses with considerable emphasis on understanding the evolution of aposematic coloration (Guilford 1992; Rowe 1999; Ruxton et al. 2004; Miller and Bee 2012; Skelhorn et al. 2016). In many prey taxa, aposematic color patterns (often employing striking combinations of reds, oranges, and yellows in order to warn potential predators) are paired with signals in other modalities (e.g., startling sounds, aversive odors, etc.) (reviewed in Rowe and Halpin 2013). These different signal components are thought to work together to signal unpalatability, but how predators perceive, process, and respond to these combinations is not well understood (reviewed in Rowe and Halpin 2013).

One hypothesis to explain the preponderance of multimodal warning displays in nature is that different signal components may interact synergistically such that one component of the signal (e.g., odor) may trigger a predator’s otherwise “hidden” aversion to another component of the signal (e.g., color) (Rowe and Guilford 1996). For example, in domestic chicks the presence of the common warning odor pyrazine triggers an otherwise hidden aversion to the colors red and yellow (colors typically associated with toxicity), but has no effect on responses to green prey (Rowe and Guilford 1996). Such phenomena have also been found in several other bird species feeding on a wide variety of insect prey (Marples and Roper 1996; Rowe and Guilford 1996, 1999a; Jetz et al. 2001; Lindström et al. 2001; Kelly and Marples 2004; Skelhorn and Rowe 2006); these studies offer a compelling explanation for why warning colors so often co-evolve with additional signal components, such as odors.

The diversity of non-avian predators, specifically terrestrial invertebrate predators, which feed on insects in nature is immense (Symondson et al. 2002) yet they have been largely ignored in the study of how predator psychology may shape the evolution of aposematic colors (Guilford 1992; Ruxton et al. 2004; Miller and Bee 2012). This is despite growing appreciation for the sophisticated cognitive abilities of invertebrates (Giurfa 2013). Moreover, the rich literature documenting the synergistic interactions between color and odor in communication between flowers and their insect pollinators (Leonard and Masek 2014; Yoshida et al. 2015) suggests that hidden psychological responses to color and/or odor might be widespread, but simply unrecognized in invertebrate predators.

Jumping spiders (Araneae, Salticidae) are an ideal group to examine the psychological responses to multimodal prey defenses. They comprise the largest family of spiders with more than 5900 described species (World Spider Catalog 2017), and are found on every continent except Antarctica (Maddison et al. 2008). Spiders in this family are voracious predators that feed on a wide variety of prey (Jackson and Pollard 1996), including agricultural pests (Young and Edwards 1990), and will attack prey considerably larger than themselves (Nintwig and Christian 1986). Indeed, they have been implicated in driving the evolution of color patterns in a range of arthropod prey (Mather and Roitberg 1987; Rota and Wagner 2006; Huang et al. 2011). While perhaps best known for the remarkable visual acuity in their principal eyes (Williams and McIntyre 1980; Harland and Jackson 2000) these tiny hunters also use olfaction to detect and pursue potential mating opportunities (Cross and Jackson 2009a; Nelson et al. 2012) and prey (Nelson and Jackson 2006). While color vision capabilities appear to vary across the family (e.g., see Zurek et al. 2015), there is behavioral evidence of some degree of color discrimination in several taxa (e.g., Nakamura and Yamashita 2000; Hoefler and Jakob 2006; Lim and Li 2006; Jakob et al. 2007). While most jumping spiders are thought to be most sensitive to colors in the UV and green portions of the spectrum, spiders in the genus *Habronattus* have a unique red filter pigment that allows them to achieve trichromatic vision and the ability to see and discriminate long-wavelength colors (including reds, oranges, and yellows) that are commonly used by aposematic prey (Zurek et al. 2015). Indeed, previous behavioral work with *Habronattus pyrrithrix* suggests that these spiders use the color red in both foraging and mating (Taylor and McGraw 2013; Taylor et al. 2014, 2016). This makes *Habronattus* jumping spiders particularly well suited for exploring the functions of multimodal warning displays.

Here, we ask how color and odor interact to influence predation when spiders were given choices between artificially colored prey in the presence or absence of an aversive odor. Based on a large body of previous work with avian predators, we hypothesized that the different signal components interact synergistically, such that one component of the signal (odor) may trigger a spider’s aversion to another component of the signal (color) (see Marples and Roper 1996; Rowe and Guilford 1996, 1999a; Jetz et al. 2001; Lindström et al. 2001; Kelly and Marples 2004; Skelhorn and Rowe 2006). This hypothesis led to the *a priori* prediction that spiders in the presence of an aversive odor would be more likely to avoid red prey. Moreover, if this phenomenon is unique to the color red (and other typical warning colors) as has been shown in birds (see Marples and Roper 1996; Rowe and Guilford 1996, 1999a; Jetz et al. 2001; Lindstrom et al. 2001; Kelly and Marples 2004; Skelhorn and Rowe 2006), this leads us to a second *a priori* prediction: the presence of the aversive odor should have no effect on spiders’ responses to green prey. Previous jumping spider studies have documented complex behavioral responses to cues in different modalities outside of the context of aposematism (e.g., VanderSal and Hebets 2007; Cross and Jackson 2009b; Nelson and Jackson 2014), yet this is the first study to examine potentially hidden color aversions triggered by the presence of odors that may have important implications for the evolution of multimodal warning displays.

## METHODS

### Study species

We collected *H. trimaculatus* (*n* = 84) from Ocala National Forest, FL (29.26965672, −81.68671561) and Forage Farm, Gainesville, FL (29.586624, −82.240876). Spiders were housed following methods provided elsewhere (Taylor et al. 2014), fed approximately their own mass in juvenile crickets (*Gryllodes sigillatus*) 3× per week and tested 14–52 days after being collected from the field.

### Experiment 1: red versus black choice tests in the presence/absence of odor

**Figure 1:**
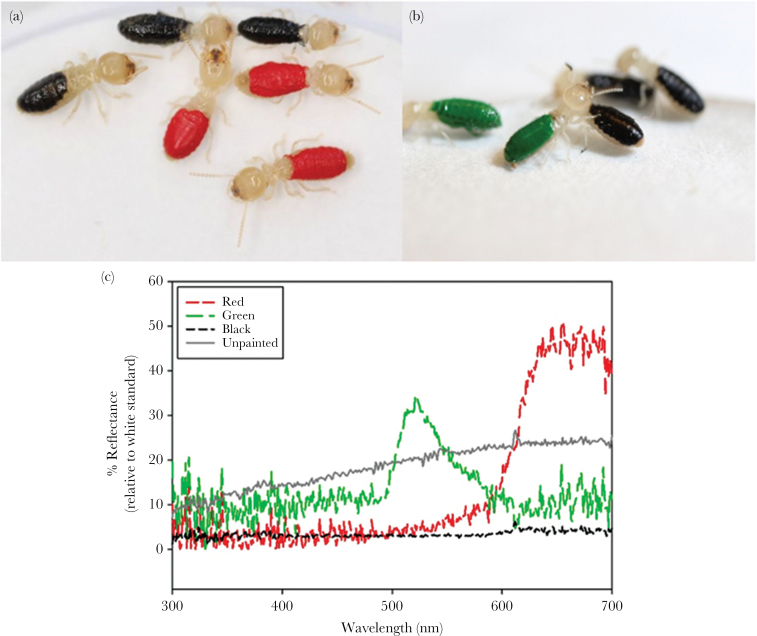
(a) Termites with abdomens painted red or black, (b) termites with abdomens painted green or black, and (c) spectral properties of artificially painted red, green, and black termites used in color choice tests. Naturally colored (unpainted) termites are also shown for comparison. Spectral curves represent the mean values for 10 individuals of each color.

We gave spiders choice tests (*n* = 42; 4 adult females, 38 immature) between color-manipulated termites (*Reticulitermes flavipes*) who had the dorsal surface of their abdomens painted with either red or black enamel paint (Testor Corporation, Rockford, IL, red: 1150-RM11501-0611, black: 1149-RM11491-0611) (Figure 1a). We used the tip of a toothpick to fully cover each termite’s dorsal abdomen with paint (Figure 1a). Spectral properties of artificially-painted termites were measured using a UV-vis spectrophotometer (USB 2000 with PX-2 pulsed xenon light source, Ocean Optics, Dunedin, FL) that can precisely measure colored areas as small as 1mm (Figure 1c). To record spectral measurements of the painted termites, we freeze-killed 10 individuals of each color and positioned the spectrophotometer probe perpendicular to the colored surface using a measurement pin attached to the probe to ensure a consistent distance between the probe and sample. Measurements were taken relative to a Spectralon diffuse reflectance white standard (Labsphere Inc., North Sutton, NH). Behavioral tests confirmed that the colored enamel did not affect the behavior (i.e., movement rates) of painted termites compared with unpainted controls (ANOVA, *F*_2,56_ = 0.07, *P* = 0.93). When dry, the enamel paint does not emit any noticeable odor; however, all termites presented to spiders during choice tests were painted (either red or black) and thus any small amount of residual paint odor should not drive any differences between our treatment groups.

To determine how the presence of an aversive odor affects color preferences during prey choice tests, half of the spiders were chosen at random to be exposed to the defensive chemicals from adult coreid bugs, *Acanthocephala femorata* (Hemiptera, Coreidae), during their color preference tests, while the other half were not exposed to these chemicals (control group). To collect these chemicals from the bugs, bugs were placed individually inside enclosed petri dishes lined with filter paper and shaken for 10 seconds to trigger the release of defensive chemicals onto the filter paper. Because previous work with avian predators has shown that novel odors are most likely to trigger color aversions compared with familiar odors (see Rowe and Guilford 1996, 1999b; Marples and Roper 1996; Jetz et al. 2001) we chose an odor that would be likely to be novel to our experimental spiders. We have never observed *A. femorata* at the field site where our spiders were collected. Moreover, adult *A. femorata* range in size from 25 to 28 mm (pers. obs.), which is 4–5× larger than adult *H. trimaculatus*. As such, if the spiders were to encounter these adult coreids in the field, they would be unlikely to elicit a chemical defense. While spiders may have had experience attacking juvenile coreids in the field, the defensive chemicals of juveniles are distinctly different than those of the adults (Aldrich and Yonke 1975; Prestwich 1976). Taken together, this suggests that adult *A. femorata* defensive chemicals represent a novel aversive odor that our experimental spiders would not have previously experienced.

**Figure 2:**
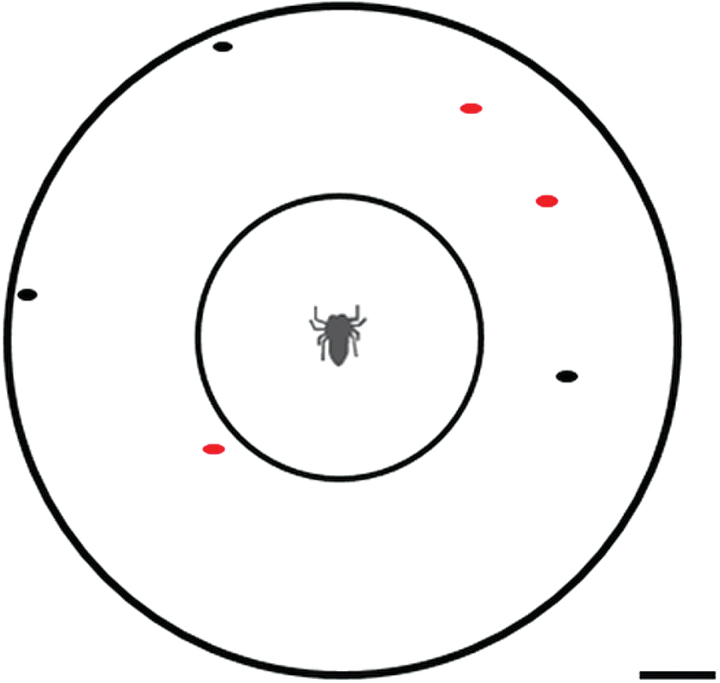
Testing arena used for choice tests (scale bar represents 1 cm). Spiders were released from the central chamber and allowed to choose from the surrounding artificially colored termites.

For the color choice tests, we presented spiders with 3 red and 3 black termites to choose among in a 9-cm diameter testing arena (Figure 2). Before starting their tests, spiders were given 10 min to acclimate in a clear central chamber lined with filter paper. For spiders in the odor treatment group, this filter paper contained coreid bug defensive chemicals (collected onto the filter paper as described above and immediately placed into the chamber at the start of the test), while spiders in the control group had filter paper with no chemicals. The filter papers were kept in the testing arena for the duration of the choice tests. Because the floor of the acclimation chamber and arena were covered with white filter paper, this provided a consistent visual background (as well as an absorbent substrate for introducing the odor).

After acclimation, spiders were released and given 10 min to attack a termite. We observed the spiders directly and recorded 1) the time it took them to orient to the first termite (salticids detect movement with their secondary eyes, but always clearly orient their bodies and their large forward-facing principal eyes towards prey or other objects that get their attention [see Harland and Jackson 2000)], 2) the color of the first termite that the spider oriented to, 3) the time it took spiders to attack their first termite, and 4) the color of the first termite attacked. For termites that were attacked, we also recorded the “evaluation time” as the time between orienting to a particular termite and attacking it. The trial ended as soon as the first termite was attacked, or if the spider did not attack any termites within 10 min. Because the termites are palatable prey for the spiders, all of those that were attacked were also quickly consumed by the spider (i.e., none were rejected). Most spiders (24/42) attacked a termite in their first 10-min trial (when the odor was presumed to be novel). Spiders that did not attack during their first trial were placed on a reduced diet (1/4 of their body size in crickets 3× per week) and were retested in the same way up to 4 times until they successfully made a choice.

Because color vision in *Habronattus* appears to be light-limited (Taylor and McGraw 2013; Zurek et al. 2015), we ran all tests in the laboratory in an area adjacent to 2 large corner windows (1.5 × 0.9 m and 2.8 × 5.0 m). All tests were completed on sunny days between 9:00 and 18:00 h, when the testing arena was fully illuminated with natural sunlight. Spiders were tested prior to being fed (on their regularly scheduled feeding day) meaning that they had not eaten for 2 days. This feeding regime was strategically chosen to result in test spiders that were usually hungry enough to attack termites in our choice tests, but that were not so hungry that they would attack the first prey item encountered (without being choosy). *H. trimaculatus* are ground-dwelling generalist predators that will readily attack most small arthropods that they encounter in the field, including termites (personal observation).

### Experiment 2: green versus black choice tests in the presence/absence of odor

In Experiment 1, we found that, as predicted by our hypothesis, spiders were more likely to avoid red prey in the presence of the novel aversive odor (see Results). These data suggest that odor can trigger an otherwise hidden aversion to a warning color (in this case, red). However, data from Experiment 1 did not allow us to discern if this finding is unique to the color red (a common warning color), or, alternatively, if the same odor would trigger an aversion to any color that a spider is presented with, even if it is not typically used as a warning (e.g., green, brown, etc.). To rule out this possibility, we performed a nearly identical experiment (Experiment 2) where we gave a new group of field-collected spiders (*n* = 42; 11 adult females, 11 adult males, and 20 immature) the choice between green and black termites (with or without the presence of coreid bug odor) (Figure 1b). The green termites used in Experiment 2 were designed to be similar in brightness to the red termites in Experiment 1; as such, any differences between the outcomes of the 2 experiments can likely be attributed to chromatic rather than achromatic (i.e., brightness) differences between the colors. To obtain a green paint that was equivalent in brightness to the red paint used in Experiment 1, we mixed white and green enamel paint (Testor Corporation, Rockford, IL, white: 1168-RM11681-0611, green: 1124-RM11241-0611), and measured the spectral properties. This final green paint mixture did not differ in mean brightness from the red (*t*_420_ = 0.12, *P* = 0.45, see also Figure 1c).

### Statistical analyses

Our basic data analysis from Experiments 1 and 2 were identical. To determine if the presence of coreid bug defensive odor altered spider color preferences, we performed likelihood ratio χ^2^ tests to compare the color of the termite attacked in a trial (red vs. black, green vs. black) by spiders in the presence vs. absence of the odor. First, we ran this analysis on all of the spiders that successfully attacked a termite during their first test. During the first round of tests, we presumed that the odor was completely novel to the spiders (see rational above). When we did find differences between the treatment groups (as was the case in Experiment 1), we went on to assess whether there was evidence of color preferences or aversions within each group using likelihood ratio χ^2^ tests (to assess whether the attack rates on the 2 different colors differed from 50/50).

If spiders did not capture a termite in their first test (i.e., chose not to attack any termites in the allotted time), they were retested the same way up to 4 times until they successfully attacked a termite. In these repeat tests, the odor was no longer novel to the spiders (i.e., they experienced the same odor again in each test). Because previous work with avian predators suggests that familiar odors are less likely to trigger color aversions compared with novel odors (see Marples and Roper 1996; Rowe and Guilford 1996, 1999b; Jetz et al. 2001), we analyzed repeat tests in a separate analysis. Because samples sizes were too low to run likelihood ratio χ^2^ tests, we analyzed the results of these repeat tests using a two-tailed Fisher’s exact test.

In Experiment 1, we found that the presence of the coreid bug odor made spiders less likely to attack red prey compared with when the odor was absent (see Results). This finding led us to conduct some exploratory *post hoc* analyses specifically on the data from Experiment 1 to help us better interpret this result. Prey survival can be accomplished by avoiding attack after being detected or, alternatively, by avoiding detection altogether. As such, one possibility is that in Experiment 1, the presence or absence of the odor may have simply influenced the color that first got a spider’s attention. To test this idea, we used likelihood ratio χ^2^ to determine if the presence of the odor affected which color the spider first oriented to. Additionally, we used ANOVA to determine whether the presence of the odor influenced how long it took the spider to orient to the prey, the latency to attack the prey, or the evaluation time (the difference in time between orientation and attacking the prey). Finally, we tested whether the presence of the odor affected whether or not the test spiders initially attacked a prey item. The goal of these additional exploratory analyses was to provide insight into how the presence or absence of the odor may have altered foraging behavior.

To rule out the possibility that differences in hunger level or predatory motivation were driving different responses between first and subsequent round tests in Experiment 1, we used a *t*-test to compare the spiders’ latency to attack prey between first and repeat tests. Hungrier spiders will usually attack prey more quickly; as such, if there are no differences in latency to attack between the 2 groups, this supports the idea that any differences are not likely to be explained by hunger level.

Finally, in both Experiments 1 and 2, we tested whether the size or the sex/stage of the spider (i.e., whether the spider was an adult female, adult male, or juvenile) had any effect on color preferences. Because the sexes look the same until sexual maturity in *H. trimaculatus* (as in many other jumping spiders), we followed the precedent in the salticid literature of grouping spiders into these 3 categories for analysis (Lim and Li 2006; Bartos 2008; see Taylor et al. 2014). Previous work with *Habronattus* jumping spiders has indicated that sexes and life stages do not differ in color preferences (Taylor et al. 2014); as such, we had no a priori reason to expect differences, but we ran these tests to confirm that that was the case in the present study.

All analyses were conducted using JMP Pro (version 12.0.1).

## RESULTS

### Experiment 1: red versus black choice tests in the presence/absence of odor

**Figure 3:**
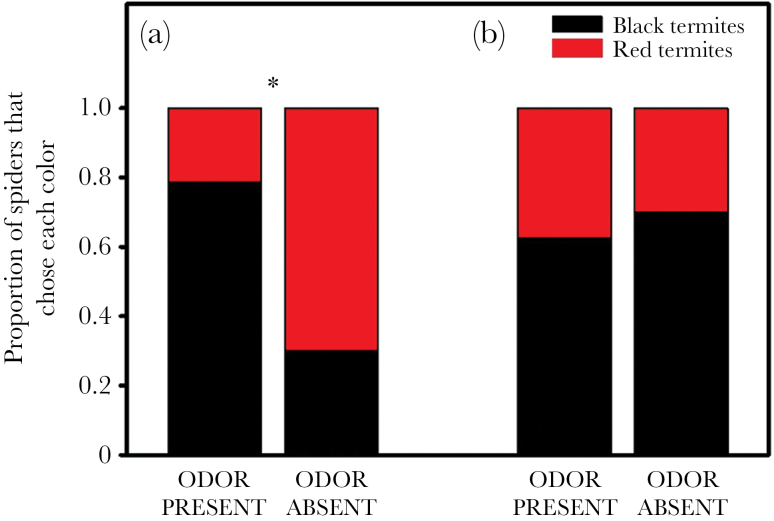
Results of choice tests between red- and black-painted termites in the presence or absence of coreid bug defensive odor. In the first round of tests (a) the odor was presumed to be novel to the spiders, while in subsequent rounds (b) the odor was no longer novel. An asterisk indicates a significant difference between treatments.

In the first round of red versus black choice tests, when the odor was presumed to be novel (*n* = 24), spiders were more likely to avoid attacking red termites when the odor was present than when the odor was absent (χ^2^ = 5.84, *P* = 0.02; Figure 3a). When the odor was absent, there was no evidence of a color bias (i.e., attacks on red vs. black did not differ from 50/50; χ^2^ = 1.65, *P* = 0.20), but when the odor was present, the spiders biased their attacks away from red (χ^2^ = 4.86, *P* = 0.03). Interestingly, in subsequent tests (*n* = 18), after the spiders had been exposed to the odor over several trials, the odor had no effect on color preferences (Fisher’s exact test, *P* = 0.99 Figure 3b).

While the presence of the odor affected which color was attacked, it did not affect the color to first capture the spiders’ attention (i.e., which color the spider first oriented to during the trial) (first round: χ^2^ = 0.97, *P* = 0.33; subsequent rounds: Fisher’s exact test, *P* = 0.32). Additionally, the presence of the odor did not affect the time it took spiders to orient to (first round: *F*_1,22_ = 0.12, *P* = 0.74; subsequent rounds: *F*_1,16_ = 1.12, *P* = 0.31) or attack the termites (first round: *F*_1,22_ = 0.09, *P* = 0.76; subsequent rounds: *F*_1,16_ = 0.35, *P* = 0.56). The time spiders spent evaluating prey before attacking was not affected by the presence of the odor (first round: *F*_1,22_ = 0.04, *P* = 0.85; subsequent rounds: *F*_1,16_ = 0.05, *P* = 0.82). Finally, the presence of the odor did not affect whether or not the spiders initially attacked a termite (χ^2^ = 0.80, *P* = 0.37).

Differences in hunger level between the first and subsequent tests likely did not influence the responses as these groups did not differ in their time to attack prey (*t*_40_ = 0.40, *P* = 0.69); this suggests that spiders on a reduced diet were not attacking prey indiscriminately due to higher hunger levels.

As in previous studies of *Habronattus* jumping spiders (Taylor et al. 2014) the sex/stage of the spider had no effect on color preferences in Experiment 1 (χ^2^ = 2.50, *P* = 0.11), nor did the size of the spider have any effect on color preferences (χ^2^ = 0.31, *P* = 0.57).

### Experiment 2: green versus black choice tests in the presence/absence of odor

**Figure 4:**
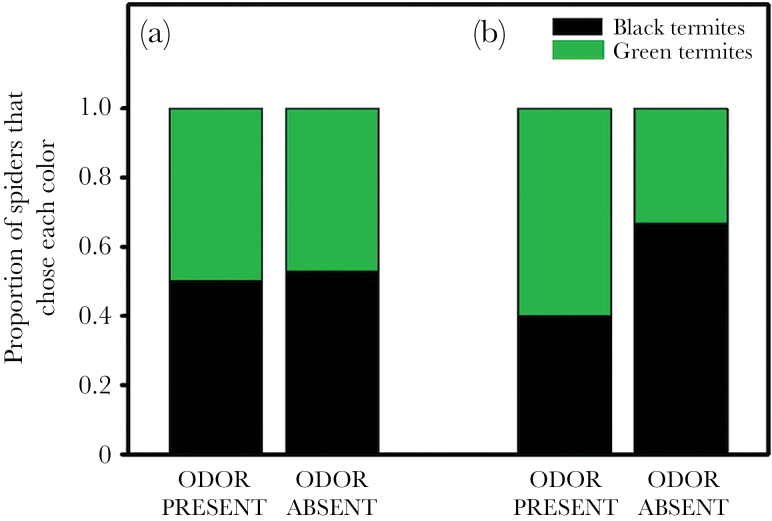
Results of choice tests between green- and black-painted termites in the presence or absence of coreid bug defensive odor. In the first round of tests, (a) the odor was presumed to be novel to the spiders, while in subsequent rounds (b) the odor was no longer novel. An asterisk indicates a significant difference between treatments.

In the first round of green versus black choice tests, when the odor was presumed to be novel to the spiders (*n* = 29), the presence or absence of the odor had no effect on color preferences (χ^2^ = 0.02, *P* = 0.90; Figure 4a). Additionally, when the odor was presumed to no longer be novel in repeat tests (*n* = 13), the presence or absence of the odor again had no effect on color preferences (Fisher’s exact test, *P* = 0.56; Figure 4b).

Unlike in Experiment 1, we did find an effect of both sex/stage and size of the spiders on their color preferences in Experiment 2 (sex/stage: χ^2^ = 11.93, *P* = 0.003, size: χ^2^ = 5.73, *P* = 0.03).

## DISCUSSION

Here, we show that the presence of an aversive odor from a coreid bug made jumping spiders more likely to avoid red prey (compared to when the odor was absent). Our results were consistent with *a priori* expectations based on a large body of work in avian predators showing that aversive odors trigger otherwise hidden aversions to aposematic colors, such as the color red (Marples and Roper 1996; Rowe and Guilford 1996, 1999a; Jetz et al. 2001; Lindstrom et al. 2001; Kelly and Marples 2004; Skelhorn and Rowe 2006). An aversive odor may indicate to a predator that chemically defended prey is in the vicinity allowing the predator to bias its attacks away from prey that are most likely to be dangerous. Interestingly, the aversive odor in our study had no effect on how the spiders responded to green prey (a color that is not typically used as a warning color); again, this is consistent with findings in avian predators (see Rowe and Guilford 1996, 1999b; Jetz et al. 2001; Rowe and Skelhorn 2005). To our knowledge, this is the first study to examine how odor may influence color preferences in an invertebrate predator, and suggests that the rules that generalist predators use to make basic foraging decisions may be surprisingly similar across a wide range of animal taxa (ranging from birds to spiders).

Our study also suggests that the novelty of a signal component may influence its role in a multimodal warning display. In the first round of tests in Experiment 1 (while the odor was presumed to be novel to the spiders), we found that the presence of odor made spiders less likely to attack red prey. Interestingly, when we retested spiders that had failed their first round of tests (i.e., they did not attack any prey in the first round), the presence of the odor no longer had any effect on their color preferences. There is a precedent in the avian literature for such a phenomenon where odors only influence color preference when they are novel (Marples and Roper 1996; Rowe and Guilford 1996; Jetz et al. 2001). In particular, Jetz et al. (2001) reported that in domestic chicks, a novel odor triggered avoidance of red and yellow prey in early experimental sessions, but by the third round of exposure, the color aversion disappeared. In our study, the odor only affected color preferences during the spiders’ first experience with it. Subtle differences in how these spiders respond to novelty (compared with birds) has important implications for how multimodal signals should evolve in different types of prey. It is important to note that our experiment was not designed to test for the effects of novelty *per se*. Instead, our goal was simply to examine the effect of a novel odor on prey color preferences. Because a number of our spiders in Experiment 1 (18/42) unexpectedly failed their first round of tests, we decided to go on and retest them even though the odor was no longer novel. This allowed us to compare responses between when the odor was novel and when it was not; however, we can’t rule out the possibility that there were other differences between these 2 groups of spiders. Future studies should examine the effect of novelty more directly by randomly assigning spiders to be exposed to either familiar or novel odors and examining how this affects their responses to the color red.

Our study shows that odor alters the color of prey items that jumping spiders attack, but more work is still needed to understand more specifically how odor and color are interacting. Prey survival can be accomplished by avoiding attack after being detected or, alternatively, by avoiding detection altogether. One possibility that we considered is that the presence of the odor may influence how the colors are perceived (see Epple and Herz 1999; Pauli et al. 1999). In our study, we found no evidence that the odor had any effect on which color first captured the spiders’ attention; it only had an effect on which color was attacked (see Results). This suggests that the odor is likely triggering either an innate aversion to the color red or, alternatively, that the odor is triggering a memory of previous negative experience with the color red (e.g., Rothschild and Moore 1987; Siddall and Marples 2008, reviewed in Rowe and Halpin 2013). Prey color biases in foraging *Habronattus* are likely made up of both innate and learned components (see Taylor et al. 2014, 2016), making either or both of these possibilities feasible. Clearly, more work is needed to understand the specific mechanisms underlying the patterns observed in our study.

Beyond the context of aposematism, there is evidence that other jumping spiders have complex behavioral responses when presented with multimodal cues. For example, *Evarcha culicivora* is a jumping spider that specializes on blood-filled mosquitoes and uses both odor and visual cues to find mosquitoes; interestingly, the presence of mosquito odor during experiments improves the spiders’ ability to find a cryptic visual mosquito lure (Cross and Jackson 2009b). This may be similar to what occurred in our experiment where the detection of an ecologically relevant odor primes the spider to attend to and respond to a particular visual cue (e.g., a mosquito image in *Evarcha* or the color red in our study). Cues in other modalities may also prime spiders to pay particular attention to color; VanderSal and Hebets (2007) found that the presence of a vibration improved jumping spiders’ ability to learn an experimental color discrimination task. Here again, this may be an example of one cue (in this case, vibration) priming the spiders to attend to color and thus improving their ability to learn and remember the task.

The field of avian predator psychology has provided immense insight into the evolution of aposematism and other prey defenses (Guilford 1992; Ruxton et al. 2004; Miller and Bee 2012; Rowe and Halpin 2013; Skelhorn et al. 2016). However, birds make up only a subset of the diversity of visual predators in nature (Land and Nilsson 2012). Spiders and other invertebrates exert extreme predation pressures on insect prey (Wise 1995), and are thus likely important in shaping multimodal prey defenses. Here we show that spiders, with vastly different sensory systems than birds (Land and Nilsson 2012), and miniscule brains (Eberhard 2011), may show remarkable similarities in their responses to multimodal signals. More work is needed to understand how different signal components interact to influence predation in other understudied predators and how these predators, collectively, have shaped the diversity of multimodal defensive strategies in nature.

## FUNDING

This work was supported by funding from a National Science Foundation grant (IOS-1557867 to L.A.T.), the Florida Museum of Natural History, and the Entomology and Nematology Department at the University of Florida. Publication of this article was funded in part by the University of Florida Open Access Publishing Fund.
